# Emerging Trends in Telehealth and AI-Driven Approaches for Obesity Management: A New Perspective

**DOI:** 10.21315/mjms-08-2024-614

**Published:** 2025-02-28

**Authors:** Thai Hau Koo, Xue Bin Leong, Jet Kwan Ng, Nicholas Kay Beng Tan, Mafauzy Mohamed

**Affiliations:** 1 Division of Endocrinology, Department of Internal Medicine, Hospital Universiti Sains Malaysia, Kelantan, Malaysia; 2 School of Medical Sciences, Universiti Sains Malaysia, Health Campus, Kelantan, Malaysia; 3 InnoDev Digital Enterprise, Kuala Lumpur, Malaysia

**Keywords:** telehealth, technology, obesity, interventions

## Abstract

One in eight people globally, prevalent mainly in some ethnic groups and those from low socioeconomic backgrounds, is a critical global health challenge of obesity. The telehealth system would be valuable in arresting obesity, with better access to care and a supporting digital community that encourages regular practice of physical activities, healthy diets, and health monitoring, all within reach and at affordable prices. Digital health interventions such as telehealth, mHealth applications, and wearable devices are new modalities for the treatment of obesity that increase monitoring of energy expenditure, physical activity level, and caloric intake. These technologies enhance the possibilities of therapy against barriers, such as maintaining motivation and improving the diet. This review summarises current evidence regarding digital health interventions for obesity management by considering and evaluating various global digital strategies to reduce obesity. The literature emphasises the effectiveness of eHealth interventions toward weight loss and maintenance. Furthermore, artificial intelligence (AI) and Information and Communications Technology (ICT) can predict and prevent paediatric obesity. By contrast, virtual reality (VR) applications can determine real-world behaviour in clinical practice. These digital interventions could increase the reach and efficacy of traditional weight management programmes by becoming more embedded in clinical practice. However, because of their broad implementation across different clinical settings, concerns regarding the security and privacy of these technologies must be addressed.

## Introduction

Obesity is conventionally defined as excess body fat, causing prejudice to health. It is usually assessed in clinical practice using the Body Mass Index (BMI), expressed as the ratio of body weight in kilograms divided by height in square metres (kg/m^2^) (1). On the national level, obesity is still viewed as a sign of personal moral failure, and obesity is not categorised as a disease (2). It is also documented that obesity affects a disproportionately higher number of certain racial/ethnic groups and lower socioeconomic and educational status (3). The worldwide prevalence of overweight and obesity is also increasing (4, 5). In 2022, 1 in 8 people were living with obesity (6), with 2.5 billion adults aged 18 years and older will be overweight, including over 890 million adults living with obesity (6). In 2019 alone, 5 million deaths from non-communicable diseases (NCDs), cardiovascular diseases (CVDs), type 2 diabetes mellitus (T2DM), cancers, neurological disorders, chronic respiratory diseases, and digestive disorders were reported from higher-than-optimal BMI (7). Hence, obesity should be addressed in future studies.

The World Health Organization (WHO) defines digital health as “the use of digital technologies for health purposes” and refers to the increasing application of technologies for health services (8). Telehealth is the most straightforward form of engagement in eHealth. This is realised through the use of telecommunications and virtual technology, providing healthcare outside of traditional settings (8). Obesity is a social rather than an individual responsibility; therefore, its remedies lie in creating supportive environments and communities that embed healthy diets and regular physical activity as the most accessible, available, and affordable behaviours in daily life (6).

Artificial intelligence (AI) in obesity management thus becomes transformative at the level of predictive analytics. AI algorithms have been applied to vast amounts of data to construct and forecast the development of obesity, considering genetics, lifestyle, and behavioural patterns. Based on this prediction, doctors can implement effective timely interventions and preventive measures to reduce the spread of the disease. These predictions also indicate that predictive analytics can make a significant difference in foresight into health outcomes, thus underlining the importance of incorporating AI into the general strategy of managing obesity.

In this review, we aimed to explore the current evidence on telehealth and technology-based interventions in the management and synthesis of obesity.

## Overview of Telehealth and AI-based Interventions

Obesity management may include various types of digital health interventions such as telehealth, mobile health (mHealth) applications, wearable devices, and online and virtual interventions. According to WHO, eHealth refers to the use of information and communication technologies for health, and mHealth refers to mobile wireless technologies for health, such as cell phones. Wearable devices can be used to monitor the energy expenditure and physical activity. Monitoring of calorie intake can be done with the aid of mHealth applications like “HealthifyMe”.

In 2010, the American Heart Association published a Society of Behavioral Medicine (SBM)-endorsed systematic review of eHealth interventions for weight loss and maintenance from 2002 to 2010 that included all types of technology, such as technology text messages, mobile devices, web-based interventions, etc. (9). In turn, improvements in digital technology allow for the automation of monitoring and feedback, thus making them more efficient and less labour-intensive for patients desiring to lose weight and the professionals in charge of the weight loss programmes (10). It has been shown that over the past decade, traditional weight management programmes have improved through the use of digital technologies or mobile applications to improve treatment adherence, recognising that a lack of maintained motivation and self-efficacy—the belief in one’s ability to undertake the intervention—and a lack of adherence to behavioural regimes are barriers to successful weight loss (11).

## Efficacy of Telehealth and AI-Based Interventions

### Weight Reducing Efficiency

The most impressive weight loss results have been observed in telehealth- and technology-based interventions, which include personal counselling or coaching by credentialed practitioners, in combination with a digital programme (12). The variable findings of the research indicate that rigorously designed future studies need to consider what is known to have an effect on the outcome measures taken (13). Access to treatment can be extended to groups that are poorly accessible by telemedicine interventions, thus offering a safe, remotely delivered alternative (14).

Remote monitoring of AI-enabled tools has changed the management of obesity by enabling the surveillance of physical activity, caloric intake, and other critical health parameters in real-time. They would present the user with ongoing assessments, which could then be sent to a healthcare provider in real-time to offer better and more relevant services. Remote monitoring allows for the improvement of telehealth interventions and guarantees that patients will receive continuing care, even from a distance. This benefit is targeted towards people living in remote or underserved areas, where access to healthcare may be limited.

### Cognitive and Behavioural Modifications

Data analysis using AI from electronic medical records will help us better understand the complexity of obesity and improve patient assessment in the future for the precise management of obesity (15). The application of telemedicine for the treatment of obesity has resulted in significant clinical improvements in nutrition, physical activity, and body weight. It offers effective remote support for individual needs and promotes sustainable health improvements (14). AI health coaches are well accepted by participants and induce behavioural changes (16). The wearable devices can track data estimating the risk of becoming obese or overweight, quantify the current level of activity, and propose a plan of intake concerning calories that is tailored to the collected information (17).

AI’s ability in extracting behavioural insights from data collected through wearables and health apps plays a major role in the management of obesity. Thus, AI continues to monitor behaviour patterns and provides real-time feedback that helps the user remain motivated to make healthier choices. Such real-time interventions are important for dealing with common barriers to weight loss, such as maintaining motivation and adherence to prescribed regimens. AI application in behavioural insights is a potent means of improving long-term health.

AI-powered virtual health coaching is a new frontier in obesity management. AI chatbots and virtual coaches are powered to provide personalised support, answer questions, and provide encouragement based on the information provided by users in terms of their interaction and progress. Thus, it simulates how human interactions deliver benefits while offering the convenience of the 24/7 availability. AI-powered health coaches have the reasonable prospect of significantly improving patient adherence to weight management programmes by providing continuous engagement and motivation, and they would be an invaluable addendum to telehealth services in this context.

### Long-Term Sustainability

The percentage of individuals attaining clinically significant long-term weight reduction can be increased, and weight loss maintenance can be improved by providing extended care through individual telephone counselling and telehealth programmes (18, 19). Even with the help of technology, adherence to dietary and nutritional therapy frequently declines with time. To improve this situation, there is an extreme need for continuous engagement strategies, individualised feedback, and regular follow-up, along with improved social support (20).

## Mechanism of Telehealth and AI-Based Interventions

Therefore, most technology-based treatments for the management of obesity are founded on well-documented theories of behaviour-change and frameworks that guide their design and implementation in effectively influencing behaviours that lead to weight loss. The models at the centre are Social Cognitive Theory, Theory of Planned Behaviour, and Transtheoretical Model, all of which underline self-efficacy, goal setting, and stages of behavioural change. For example, the ENGAGED study integrated elements of the Diabetes Prevention Programme with social support, accountability, and feedback mechanisms via mobile technology, grounded in the principles of self-regulation and control theory for long-term behavioural change (21–23).

Building on these behavioural frameworks, AI-driven personalised recommendations are a hallmark of modern obesity management strategies. When interpreting all health data and individual activity levels, if not personal preferences, AI systems can generate individually tailored diets and exercise plans that are better adhered to by patients. This level of personalisation enhances patient outcomes and increases the overall effectiveness of weight management programs. AI tailoring interventions to individual patient needs is one such impressive advancement in the fight against obesity.

Interventions have also integrated elements of cognitive-behavioural therapy (CBT) into digital platforms aimed at reducing unhealthy eating habits and increasing physical activity. Some of these studies, such as those completed by Manzoni et al. (24), have associated CBT with the addition of virtual reality (VR) modules related to superior weight loss and maintenance at one year, probably due to an improvement in body image and other emotional correlates of obesity. Other studies replicated the results of Ross and Wing (25) regarding the magnitude of weight loss and increased adherence through self-monitoring applications.

Three critical elements of technology-based interventions that help in weight loss are goal setting, self-monitoring, and feedback. Setting weight loss goals provides specific targets and therefore enhances motivation and adherence. It has also been reported that more significant weight loss occurs with more goal sets, and that weight is recorded more frequently (26). Tools for self-monitoring, especially those for logging dietary intake and physical activity, are essential tools for self-monitoring. Thomas et al. (27) and Carter et al. (28) conducted studies that reported high weight loss in randomised groups using self-monitoring applications. Feedback mechanisms—either from the self-monitoring tool itself or remote coaches—emphasise and thus reinforce desirable behaviours but also provide corrective guidance that helps improve overall adherence and outcomes (29, 30).

AI can absorb all device data, electronic health records, fitness trackers, and mobile health application data, and process them into an overall picture of a patient’s health and well-being. This holistic approach helps in effectively designing management strategies for healthcare providers based on individual needs. Probably, the most outstanding contribution of AI, up to now, in the modern management of obesity was the objective of bringing a cohesive analysis framework into a unitary system with the integration of diverse data points to ensure precision and impactful interventions.

Patient engagement and adherence were the two major factors that determined the success of the interventions. Strategies to improve engagement include behavioural tailoring of feedback, social support in the form of digital communities, and motivational messages through regular reminders. Literature has reported that compared with the usual care group, those receiving text message support and coaching had higher adherence rates and greater weight loss (31). Evidence also exists regarding the effectiveness of push notifications in improving adherence to dietary patterns and physical activities (32). Periodic follow-ups further enhance long-term behavioural changes by strengthening the patient’s commitment and discussing problems (33, 34).

## Upsides and Downsides of Telehealth and AI-based Interventions

### Positives of Telehealth and AI-based Interventions

The integration of telehealth programmes into community health plans aids population health and communities (35). The adoption and utilisation of telehealth technologies have been widespread, proving to be an excellent channel towards the delivery of quality healthcare outcomes and services (36). Digital technologies can improve the efficiency of healthcare delivery, personify and optimise medicine, and improve health and well-being (37, 38).

It reduces patient travel and wait times, increases efficiency without net cost increases, provides access to resources and care for patients in remote or underserved locations, and allows equal- or superior-quality care (39). In the short-to medium-term, telehealth does not necessarily mean healthcare cost reduction for the health system. Benefits other than cost savings should drive health services by considering telehealth implementation (40).

### Challenges in Telehealth and AI-based Interventions

Other barriers to the widespread adoption and use of telemedicine include concerns about the security and privacy of telehealth technologies. Patients and providers should ensure that the information shared in the course of telehealth consultations is secure and confidential (39). Virtual physicians lack the benefit of a thorough history and physical examination to aid diagnosis and treatment, and some argue that telehealth negatively affects the continuity of care. Furthermore, online interactions have been argued to be less intimate and secure (41).

Technical support, team-based care in the telehealth setting, and communication training to decrease the decline in the relationship between the patient and provider via telemedicine should also be included as additional support and resources (42). There is a need for further research to identify which types of eHealth interventions seem to be the most effective and how best to implement them in clinical practice (43). Further planned research is necessary to evaluate this concern because the practice of telehealth raises several questions with respect to malpractice liability regarding informed consent, practice standards, protocols, supervision requirements for non-physician providers, and availability of professional liability insurance coverage (41).

## Unique Population and Equity Consideration

Obesity is a disorder that affects more than 200 million men and 300 million women worldwide, and is spreading as an epidemic in most countries (44). Several international recommendations for the management of adult obesity have been published in Europe, the United States (US), and Australia. Moreover, national rules have been developed that consider the diversity, uniqueness, and cultural distinctions of every nation (45).

Obesity during teen years, also known as teenage obesity, is a significant risk factor for a wide range of severe long-term health problems including diabetes, hypertension, heart disease, stroke, osteoarthritis, and several types of cancer. Simultaneously, this age group tends to be predisposed to obesity during adulthood. This makes the treatment of obesity in adolescents very important because 80% of obese teens become obese adults, who are at an increased risk of developing type 2 diabetes mellitus (T2DM) and cardiovascular disease (CVD). In addition, statistics show that 93% of teens in the US have a computer, and they use it mostly from home. Moreover, 47% had smartphones and a large percentage (78%) owned cell phones. Adolescents are extensive users of technology; thus, there is an opportunity for health promotion that includes the use of new media platforms integral to youth culture (46). In busy primary care clinics, mobile technology can provide feasible and reliable methods for obesity management. Wearable technologies that provide feedback on physical activity or energy expenditure may provide additional opportunities for interventions (47).

## Strengths and Limitations

The strength of this literature review is that it comprehensively reviewed a range of digital interventions and reported behaviour-change theories to explain their effectiveness. However, variability in the study design and methodology is a major limiting factor in generalising the overall results. Another major drawback to the wide-scale use of this technology is concerns regarding the data security and privacy associated with digital health. Despite these limitations, it is clear and of significant note that these technologies have potential benefits for the management of obesity epidemics.

## Future Directions and Recommendations

There has been an updated overview of the evolution of digital therapies in recent years and their efficacy in weight reduction and weight loss maintenance over time among overweight and obese individuals in 26 systemic reviews (44, 45). The developed approach showed that eHealth therapies could be successfully utilised to deliver low-cost obesity treatments on a wider scale. Further research is therefore needed to establish both, which means that the most effective types of eHealth interventions appear to be and how they can best be adopted in clinical practice.

AI-based methods have recently enabled the prediction of the occurrence of paediatric obesity as early as two years of age; thus, it will be easier to detect, prevent, and treat obesity at this age (48). Key technical components of AI-based techniques and tools will probably help to keep this paediatric population motivated, always engaged, and living comfortably at home (49).

Innovative computer-aided or mobile device-aided information and communication technologies can make a difference in creating practical ways toward intelligent digital health treatments that could potentially help reduce childhood obesity. Internet-linked systems, electronic health records, clinical/demographic information captures, and sensors/cell phones recording eating habits are all sources from which data can be inferred based on the health, behaviour, and progress of users (50).

Various studies have shown that the state experience in VR affects user behaviour in the real-world. Previous research has also shown that the behaviour of subjects in VR environments is the same as that in real-world conditions. Considering the above data, studies regarding procedures for the prevention and treatment of obesity in virtual environments could be applied in clinical practice (51).

## Conclusions

This review provides evidence-based insights into the potential of digital health interventions for effective management of obesity in large segments of the human population. A demonstration of how technologies, including telehealth, mobile health apps, and wearable devices, can enhance treatment and support for weight loss strengthens the integration of digital health solutions into conventional healthcare practices. These findings suggest that digital health interventions can increase access, efficiency, and personalisation to manage obesity. Other results from previous research in this area provide a clear advantage in healthcare with respect to technology in increasing patient engagement and improving self-monitoring of patient-reported outcomes by providing real-time feedback. Literature mentions innumerable times to indicate early prediction and prevention of obesity using AI and Information and Communications Technology (ICT).
